# The distribution characteristics of aerosol bacteria in different types of sheepfolds

**DOI:** 10.3389/fvets.2024.1348850

**Published:** 2024-02-13

**Authors:** Jiandong Wang, Youli Yu, Abdul Raheem, Yanan Guo, Qing Ma, Doukun Lu

**Affiliations:** ^1^Institute of Animal Science, NingXia Academy of Agricultural and Forestry Sciences, Yinchuan, China; ^2^National Key Laboratory of Agricultural Microbiology, Huazhong Agricultural University, Wuhan, China

**Keywords:** sheepfolds air, microbial community structure and diversity, microbial aerosols, Miseq sequencing, pathogenic bacteria

## Abstract

With the development of modern sheep raising technology, the increasing density of animals in sheep house leads to the accumulation of microbial aerosols in sheep house. It is an important prerequisite to grasp the characteristics of bacteria in aerosols in sheep house to solve the problems of air pollution and disease prevention and control in sheep house. In this study, the microorganisms present in the air of sheep houses were investigated to gain insights into the structure of bacterial communities and the prevalence of pathogenic bacteria. Samples from six sheep pens in each of three sheep farms, totaling 18, were collected in August 2022 from Ningxia province, China. A high-volume air sampler was utilized for aerosol collection within the sheep housing followed by DNA extraction for 16S rRNA sequencing. Employing high-throughput 16S rRNA sequencing technology, we conducted an in-depth analysis of microbial populations in various sheep pen air samples, enabling us to assess the community composition and diversity. The results revealed a total of 11,207 operational taxonomic units (OTUs) within the bacterial population across the air samples, encompassing 152 phyla, 298 classes, 517 orders, 853 families, 910 genera, and 482 species. Alpha diversity and beta diversity analysis indicated that differences in species diversity, evenness and coverage between different samples. At the bacterial phylum level, the dominant bacterial groups are Firmicutes, Proteobacteria, and Actinobacteria, among which Firmicutes (97.90–98.43%) is the highest. At the bacterial genus level, bacillus, Bacteroides, Fusobacterium, etc. had higher abundance, with Bacillus (85.47–89.87%) being the highest. Through an in-depth analysis of microbial diversity and a meticulous examination of pathogenic bacteria with high abundance in diverse sheep house air samples, the study provided valuable insights into the microbial diversity, abundance, and distinctive features of prevalent pathogenic bacteria in sheep house air. These findings serve as a foundation for guiding effective disease prevention and control strategies within sheep farming environments.

## Introduction

Microorganisms are widely present in nature and can form microbial aerosols when suspended in the air in a single-cell suspension state, connected to dry solid particles (dust) and liquid particles (1, 2). Air soluble stocks of environmental microorganisms in farms can cause a variety of diseases, allergies, and toxin poisoning, cause the decline of animal resistance, induce other diseases, bring huge economic losses to the aquaculture industry, and pose a serious threat to animal welfare (3, 4). In recent years, China’s sheep breeding industry has gradually changed from the scattered breeding mode to the intensive one. The number of closed sheep sheds has gradually increased, the feeding density has increased, the breeding environment has become worse, and the disease is frequent. Past investigations have underscored the adverse health effects correlated with exposure to elevated concentrations of these microbial aerosols, including but not limited to the heightened susceptibility to afflictions such as respiratory diseases, gastrointestinal diseases, and skin diseases (5–7) including the recent outbreak of novel coronavirus, which may also be transmitted through aerosols (8). The above research suggests that the risk of respiratory symptoms in sheep houses is higher compared with people in offices or normal human living environments (3).

Traditional microbial culture has shortcomings such as being time-consuming, heavy workload, overestimation of the importance of microorganisms growing on the surface of the culture medium, etc., and many microorganisms in nature are difficult to isolate and culture and obtain their pure cultures, resulting in the detection of only some microorganisms or specific pathogenic microorganisms, which has great limitations in species and quantity analysis (9). With the continuous development of high-throughput sequencing technology, 16S ribosomal RNA (rRNA) gene sequence analysis methods have been widely used to describe the diversity of bacterial communities (10). By identifying and characterizing specific bacterial species through sequencing, researchers gain invaluable insights into disease dynamics and transmission patterns. Comprehensive analysis of these microbial populations not only enhances our understanding of disease epidemiology but also informs the development of targeted preventive measures.

Sheep are an important group of commercial species raised in Ningxia Province, China. However, because of the lack of related large-scale sheep breeding enterprises, most sheep farms are still based on traditional closed breeding, and the structure of the sheep house is relatively simple. Furthermore, there is no automatic excrement cleaning or automatic control of the environment in sheep house-related equipment. During the winter, there is an increase in respiratory illness among farmworkers, as microbial aerosol concentrations tend to increase with particulate concentrations because of reduced ventilation in sheep houses (11, 12).

In this study, the concentrations of aerosol bacteria and bacterial communities, as well as bacterial community structure, pathogenic abundance and implications for livestock biosafety of sheep houses, were compared in three typical closed sheep farms in Ningxia province. Our data provide a novel basis for the threat to public health of sheep house aerosol bacteria.

## Materials and methods

### Characteristics of study subjects

In August 2022, 18 samples were collected from three representative sheep farms in Guyuan city, China. Six samples were collected from the same fence of each sheep farm. The indoor structure of the sheep house includes a steel plate roof and has ventilation and water curtains to maintain a relatively constant temperature and humidity. Each sheep house covers an area of about 75 × 15 m^2^. Every sheep house is 3 m away, and the excrement in the sheep shed is cleaned every 15 days. In the course of our investigation, no disease outbreak occurred on any farm. Samples were collected 10 days after fecal clearance.

### Microbial aerosols collection

The collection of microbial aerosols followed the methodology outlined in prior research (3). In summary, a high-volume air sampler (HH02-LS120 of Beijing Huaruihean Technology Co., Ltd., Beijing, China) equipped with 20.32 × 25.4 cm^2^ Tissuquartz filters (PALL, Port Washington, NY, United States) was utilized for aerosol collection within the sheep housing. Positioned in the central aisle of the sheep facility at a height of 60 cm above the ground. Each membrane was cut into an average of eight pieces with sterile surgical scissors, with a weight error of ±1 mg for each part. Ultrapure water (ST876, Yaji, Shanghai, China) was added to elute the particles. Homogenization was carried out with a homogenizer at 4°C at 25,000 × *g* centrifugation for 10 min. All the filters were sterilized by baking in a muffle furnace at 500°C for 48 h before sampling. The filters were stored in a mobile refrigerator, collected and transported to the laboratory. Samples were stored at −80°C until analysis.

### 16S rRNA gene sequencing and analysis

The extraction of total genomic DNA from microbial aerosol samples followed established protocols outlined in previous studies (13, 14). The bacterial Genomic DNA Extraction Kit (Tangen, Catalog No. DP302, Beijing, China) was used to obtain total DNA by the manufacturer’s protocol. The concentration and quality of the extracted DNA were assessed using a NanoDrop 2000 spectrophotometer (Thermo Fisher Scientific, Wilmington, DE, United States), ensuring the reliability of the genetic material (OD260/OD280). PCR amplification was performed after qualified detection. The unqualified samples were extracted again until they passed the test. For bacterial diversity analysis, primers 515F (5′-GTGCCAGCMGCCGCGGTAA-3′) and 806R (5′-GGACTACHVGGGTWTCTAAT-3′) were used to amplify the V3/V4 region of the 16S rRNA gene. For 16S rRNA high-throughput sequencing, the PCR products from each sample were transferred to Ningxia Baipu Bioinformatics Technology Co., Ltd. (Baipu, Ningxia, China). All original sequences were deposited in GenBank under accession number RPJNA1048858.

The results obtained from Illumina sequencing were subjected to rigorous analysis, involving the elimination of low-quality sequences, sample identification through Barcode, and subsequent removal of Barcode and primer sequences to obtain a refined sequence file. The data were processed using QIIME (V1.8.0), which facilitated the clustering of sequences into operational taxonomic units (OTU) for precise species classification. The QIIME software was employed to compute essential alpha diversity indices, including chao1 index, observed species index, shannon index, simpson index, faith’s pd. index, pielou’s evenness index, and good’s coverage index, offering a comprehensive evaluation of the microbial diversity within the samples.

## Results

### Size distribution of culturable airborne bacteria

**Figure 1:**
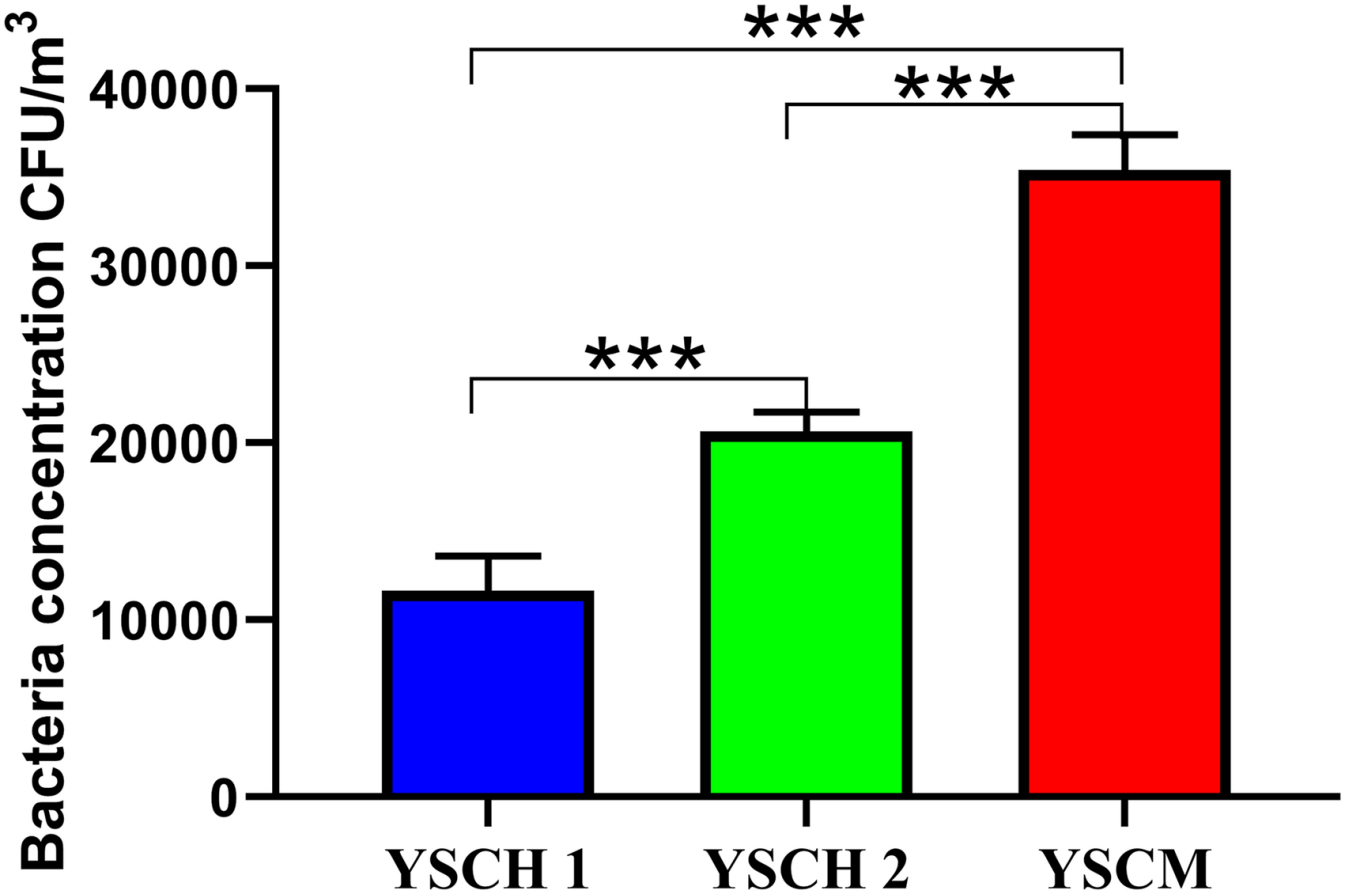
Concentrations of culturable airborne bacteria in different sheep houses (data are expressed as mean ± SD. ^***^p < 0.001). YCSH 1, YCSH 2, and YCSM represent three different sheep houses, respectively.

The concentrations of culturable bacteria in the air of the three sheep forms (YCSH1, YCSH2, and YSCM) are shown in Figure 1. The concentrations of airborne bacteria in the YCSH1, YCSH2, and YSCM were 10.235 ± 2.814 × 10^3^, 19.873 ± 1.562 × 10^3^, and 33.985 ± 2.856 × 10^3^ CFU/m^3^, respectively. Bacterial aerosol concentrations were significantly higher in the YCSH2 and YSCM than in the YCSH1 (*p* < 0.001) and significantly higher in the YSCM than in the YCSH2 (*p* < 0.001).

### Sequencing analysis

Eighteen air samples from three sheep houses (YCSH1, YCSH1, and YSCM) were sequenced for the bacterial V3-V4 region. After filtering out the sequences with poor quality, 2,004,087 valid reads were obtained. The 16S rRNA unique reads were obtained by segregating the sample sequences based on the Barcode tags. These unique reads were then clustered into Operational Taxonomic Units (OTUs) for species classification, considering a 97% similarity threshold (Supplementary Table S1). A total of 11,207 OTUs were identified in 18 samples, including 152 phyla, 298 classes, 517 orders, 853 families, 910 genera, and 482 species of bacteria (Table 1). Meanwhile, the dilution curves of all samples were infinitely close to saturation, indicating that most bacteria in the samples were detected, ensuring the reliability and stability of subsequent analysis (Supplementary Figure 1).

**Table 1:** Statistics of annotation results in species taxonomy of samples.

Filed	Sample	Phylum	Class	Order	Family	Genus	Species
YCSH 1	YCSH1_1	7	15	24	42	43	29
	YCSH1_2	9	18	26	44	50	29
	YCSH1_3	8	18	26	36	42	22
	YCSH1_4	9	18	32	45	49	29
	YCSH1_5	6	11	20	43	40	27
	YCSH1_6	9	17	29	37	41	25
YCSH 2	YCSH2_1	10	15	27	43	52	29
	YCSH2_2	11	18	30	46	43	16
	YCSH2_3	8	18	30	49	48	23
	YCSH2_4	6	15	33	54	70	28
	YCSH2_5	7	17	30	55	50	22
	YCSH2_6	9	20	33	59	52	23
YCSM	YCSM_1	12	20	37	61	75	33
	YCSM_2	9	19	35	55	61	33
	YCSM_3	8	13	25	45	49	30
	YCSM_4	8	15	30	46	42	31
	YCSM_5	10	19	29	48	45	27
	YCSM_6	6	12	21	45	58	26
	Total	152	298	517	853	910	482

### Diversity analysis of bacterial community

**Figure 2:**
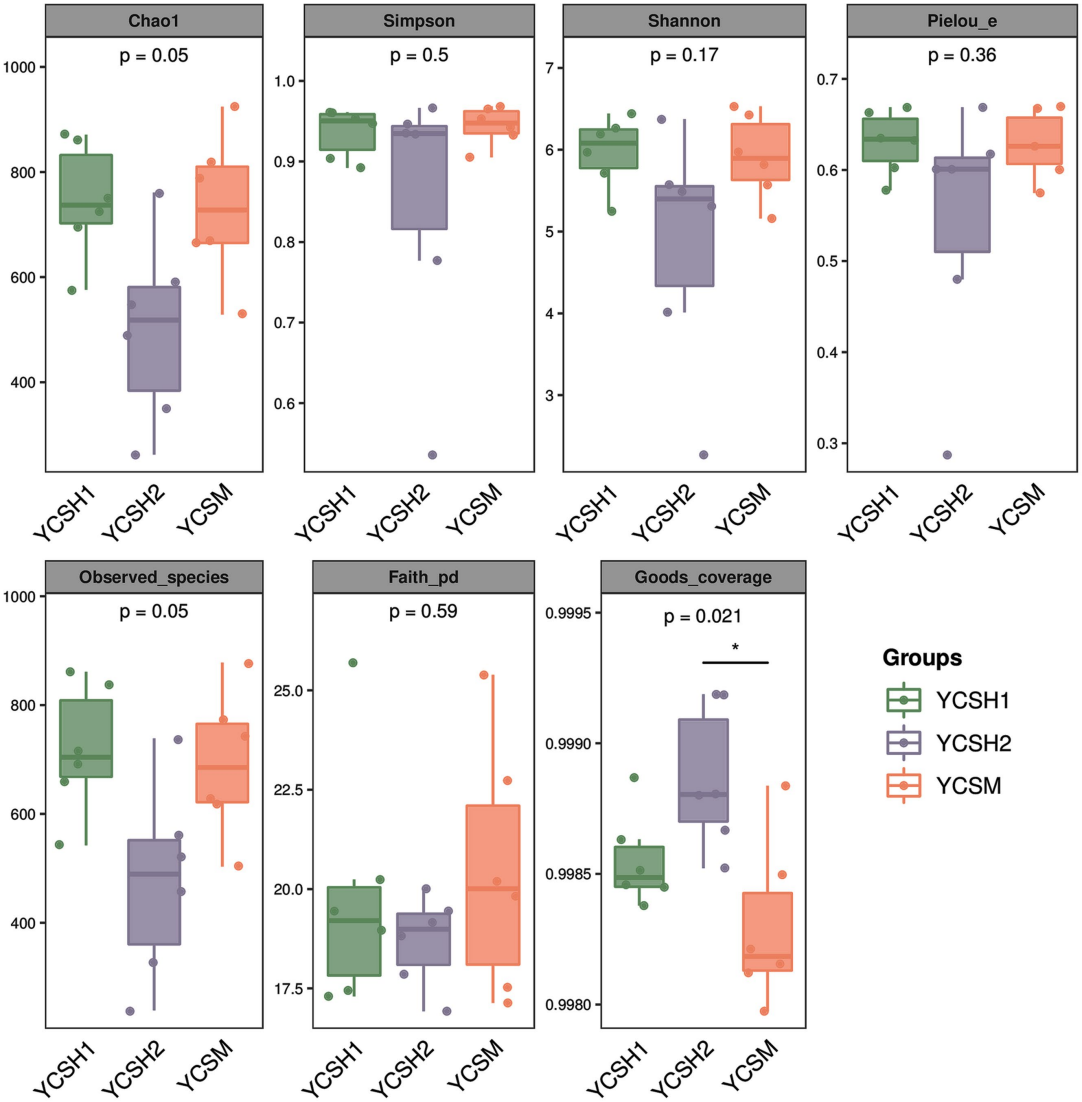
Grouped box diagram of Alpha diversity index. In each box chart, the Abscissa is the grouping label and the ordinate is the value of the corresponding alpha diversity index.

**Figure 3:**
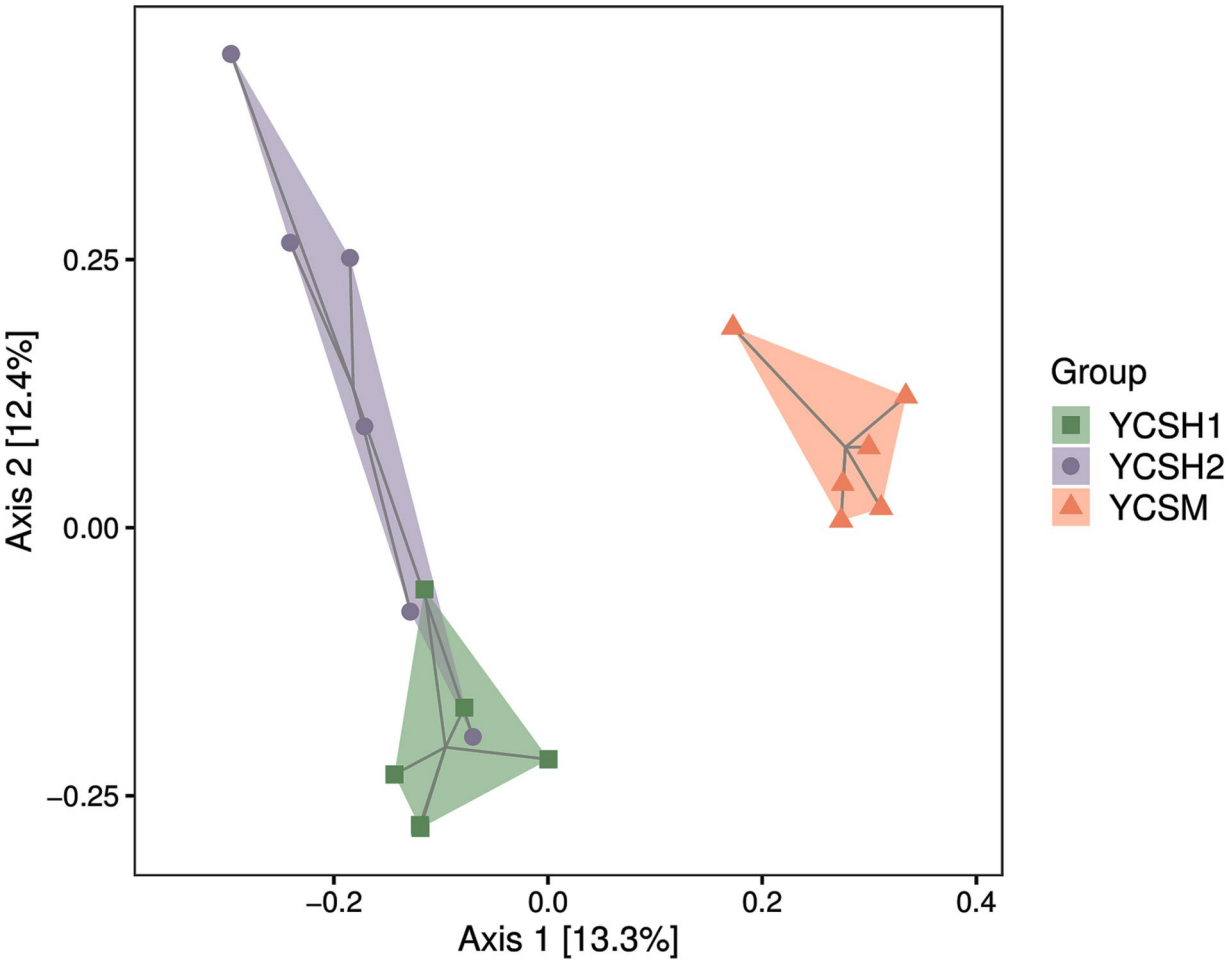
Sample two-dimensional sorting chart for PCoA analysis.

Alpha diversity, assessing species diversity within a sample, utilizes the Chao1 index for species richness prediction and the Shannon index for diversity pedigree. Higher Chao1 and Shannon values indicate increased richness and diversity, respectively. Figure 2 shows that sheepfold air samples from YCSM have the highest diversity, likely influenced by factors such as air conditions, particle levels, UV radiation, and airflow speed. Beta diversity analysis compares species differences across samples. Principal Coordinate Analysis (PCoA) in Figure 3 reveals similarities between YCSH1 and YCSH2 in spatial distribution and community proportions. However, YCSH1, YCSH2, and YCSM exhibit significant differences in spatial distribution and communities.

### Analysis of microbiota composition

**Figure 4:**
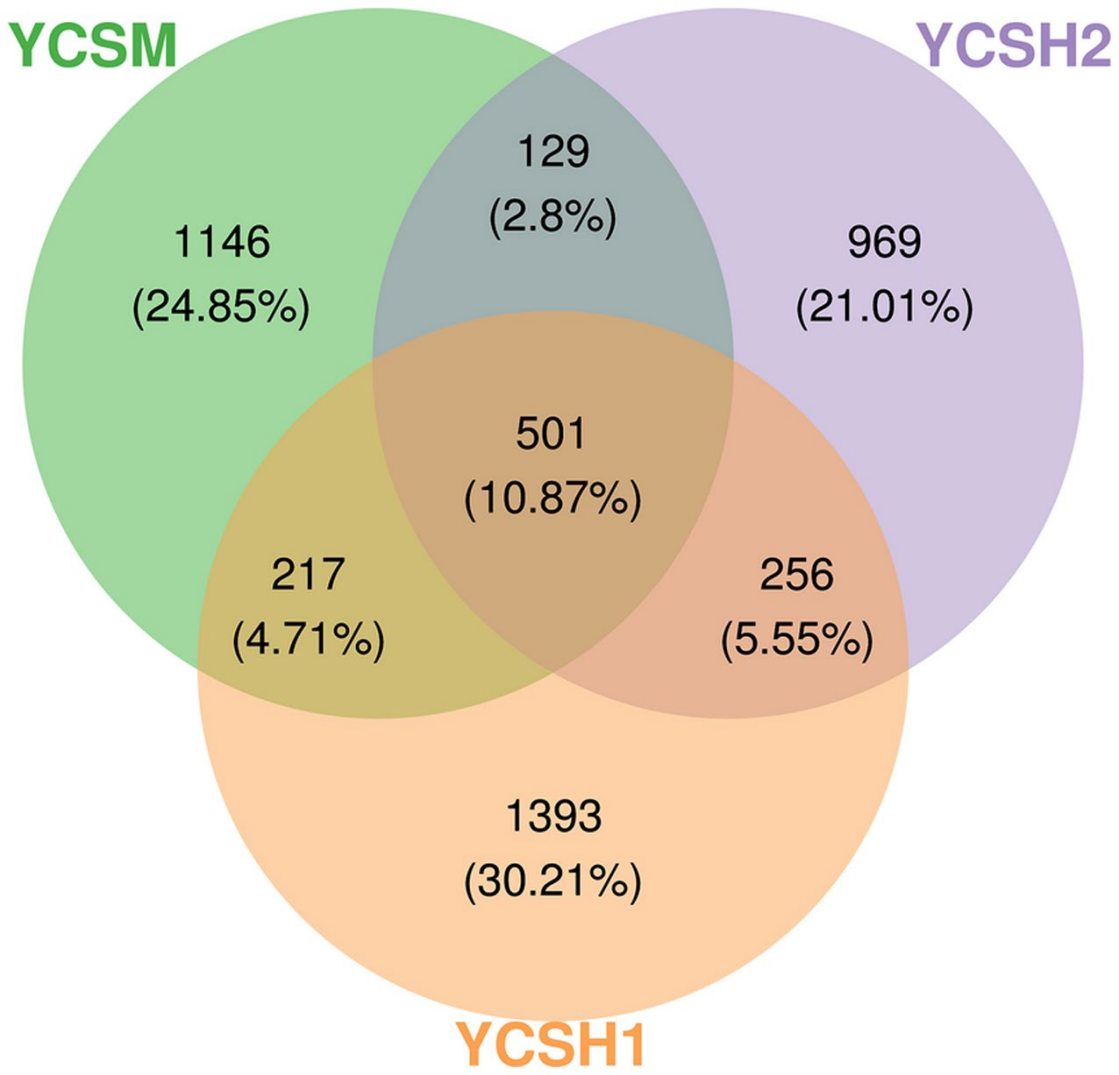
Wayne diagram of three groups of samples ASV/OTU. Each ellipse represents a group, the overlap area between ellipses indicates the common ASV/OTU between groups, and the number of each block indicates the number of ASV/OTU contained in the block.

To identify common and unique species among the 18 samples from three sheep farms, an analysis was conducted using R script and the Venn Diagram package to construct Venn diagrams for community analysis. As depicted in Figure 4, the YCSM sample comprises 1,146 species, YCSH1 includes 1,393 species, and YCSH2 contains 969 species. The YCSM group and YCSH1 group share 217 species, while the YCSM group and YCSH2 group share 129 species. Additionally, the YCSH1 group and YCSH2 group have 256 species in common. Moreover, 501 species are shared among the YCSM, YCSH1, and YCSH2 groups, providing valuable insights into the species composition across the different samples.

**Figure 5:**
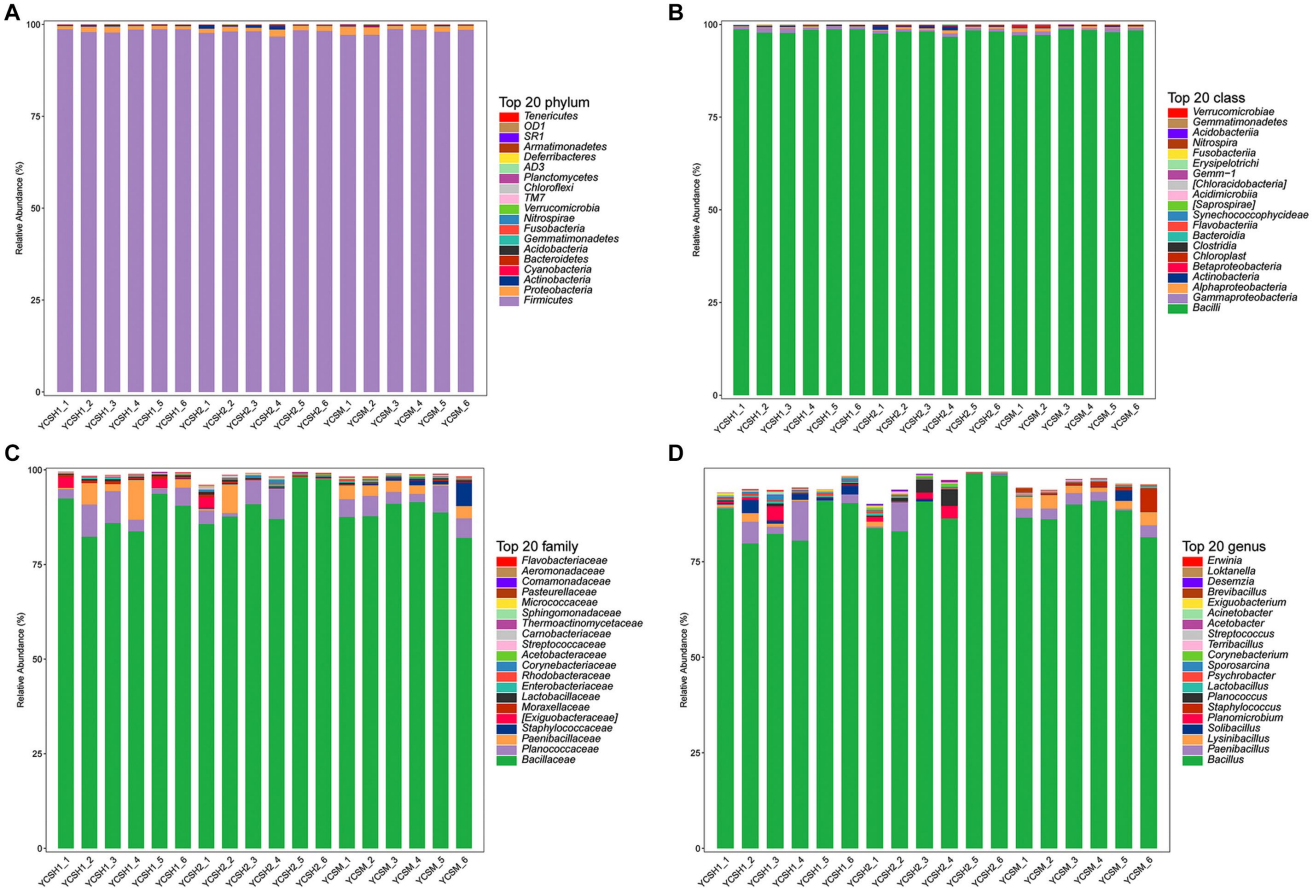
The composition of the microbiota in the sheep house air. Relative abundance of community at the phylum **(A)**, class **(B)** family **(C)**, and genus **(D)**.

At the phylum classification level, Firmicutes were most abundant. Across the 18 samples, its relative abundance ranged from 96.73 to 98.80%. Proteobacteria showed a relative dominance ranging from 0.85 to 2.30%, while Actinobacteria ranged from 0.08 to 1.00%. Conversely, bacterial groups like Cyanobacteria and Bacteroidetes exhibited relatively low proportions. The results indicate that in the air colonies of sheep pens in different farms, Firmicutes, Proteobacteria, and Actinobacteria were the dominant bacteria, whereas other bacterial groups were non-dominant (Figure 5A).

**Figure 6:**
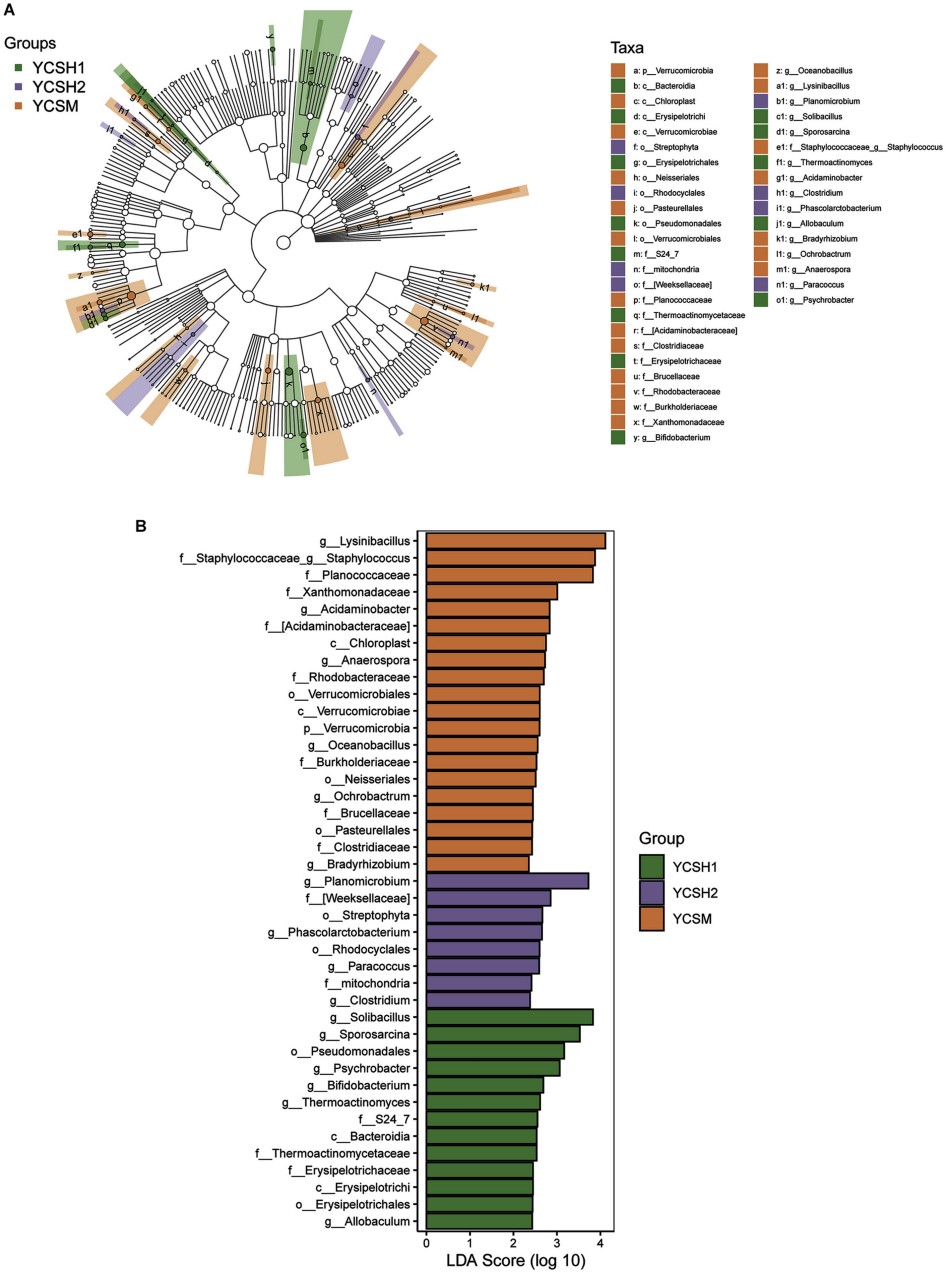
Microbiota analysis using LEfSe was conducted on sheep samples. **(A)** Linear Discriminant Analysis (LDA) effect size (LEfSe) was applied to microbial communities in distinct forms, revealing significant differences (*p* < 0.05, LDA > 3). Nodes of varying colors and sizes represent microbial groups with notable disparities and enriched species abundance. Light yellow nodes indicate groups with no significant differences. Panel **(B)** illustrates LEfSe results for three specific regions.

The dominant classes were Bacilli, Gammaproteobacteria, Alphaproteobacteria, and Actinobacteria (Figure 5B) and the dominant families were Bacillaceae, Planococcaceae, Paenibacillaceae, and Staphylococcaceae (Figure 5C). Whereas the predominant genera included Bacillus, Paenibacillus, Lysinibacillus, and Solibacillus among others (Figure 5D). Linear discriminant analysis (LDA) effect size was employed to assess the relative abundance of distinct bacterial taxa across various regions (Figure 6A). Species with LDA scores surpassing 3 were identified as significant biological markers for distinct groups (Figure 6B). The LEfSe results highlighted a varying number of significantly enriched bacterial taxa in the samples.

### Detection and analysis of high-abundance animal pathogens in air samples

In the analysis of different sheep house air samples, animal pathogens with high abundance were identified and characterized. Table 2 presents the distribution characteristics of 15 potential animal pathogens, classified under the phyla Firmicutes and Proteobacteria. Notably, Phaeophyta emerged as the dominant phylum of microorganisms in the sheep pen air. Further analysis revealed the presence of 15 animal pathogens across all 18 sheep house air samples. These pathogens include Bacillus, Psychrobacter, Acinetobacter, Exiguobacteraceae, Paenibacillus, Planococcus, Sporosarcina, Turicibacter, Streptococcus, Bifidobacterium, Thermoactinomycetaceae, Exiguobacterium, Erwinia, and Planococcaceae, among others. The clinical significance of each pathogenic bacteria is shown in Table 3.

**Table 2:** Distribution characteristics of potential animal pathogens in air samples from sheep houses.

Genus	Phylum	YCSM	YCSH 1	YCSH 2
*Bacillus*	Firmicutes	87.23%	85.47%	89.87%
*Psychrobacter*	Proteobacteria	2.34%	2.53%	1.34%
*Acinetobacter*	Proteobacteria	1.60%	1.52%	1.13%
*Exiguobacterium*	Firmicutes	1.50%	0.90%	1.03%
*Paenibacillus*	Firmicutes	0.76%	0.82%	0.75%
*Planococcus*	Firmicutes	0.60%	0.75%	0.45%
*Sporosarcina*	Firmicutes	0.53%	0.69%	0.34%
*Solibacillus*	Firmicutes	0.26%	0.60%	0.31%
*Turicibacter*	Firmicutes	0.19%	0.42%	0.31%
*Streptococcus*	Firmicutes	0.16%	0.38%	0.27%
*Bifidobacterium*	Actinobacteria	0.16%	0.32%	0.21%
*Thermoactinomycetaceae*	Firmicutes	0.12%	0.22%	0.17%
*Exiguobacterium*	Firmicutes	0.12%	0.16%	0.15%
*Erwinia*	Proteobacteria	0.11%	0.15%	0.14%
*Planococcaceae*	Firmicutes	0.09%	0.11%	0.14%

**Table 3:** The clinical significance of potentially pathogenic microorganisms in the air of sheep house.

Genus	Clinical significance	References
*Bacillus*	There are various resistance mechanisms of Bacillus. Bacillus can inhibit the growth of a variety of pathogens by secreting a large number of antibacterial secondary metabolites, such as *Bacillus licheniformis* can regulate the micro-ecological environment of the animal digestive system. Provide high-quality nutrients and a variety of bioactive enzymes conducive to digestive function for the host. Pathogenic members mainly include zoonotic pathogens such as *Bacillus anthracis* and *Bacillus cereus*	(14)
*Psychrobacter*	Psychrobacter, another prevalent genus, holds clinical significance, with certain strains causing human infections, including ocular infections, bacteremia, endocarditis, and meningitis.	(15)
*Acinetobacter*	Acinetobacter is a conditional pathogen that can cause bacteremia and pneumonia, meningitis, peritonitis, endocarditis, urinary tract, and skin infections.	(16)
*Exiguobacterium*	Diverse isolates sourced from different nich have been systematically investigated for their applications in biotechnology and industry. These applications span enzyme production, bioremediation, and the degradation of environmentally harmful substances. Notably, certain isolates exhibit plant growth-promoting capabilities, prompting ongoing exploration for their potential role in enhancing agricultural productivity.	(17)
*Paenibacillus*	Numerous Paenibacillus species exhibit direct contributions to crop growth through mechanisms such as biological nitrogen fixation, phosphate solubilization, synthesis of the phytohormone indole-3-acetic acid (IAA), and the release of siderophores facilitating iron acquisition. However, it is essential to note potential drawbacks, such as *Paenibacillus larvae* causing American Foulbrood, a fatal disease in honeybees. Additionally, certain Paenibacillus species can opportunistically infect humans, while others contribute to the spoilage of pasteurized dairy products.	(18)
*Planococcus*	The halophilic bacterium genus Planococcus is renowned for its production of various secondary metabolites. Noteworthy examples include 2-acetamido-2-deoxy-α-d-glucopyranosyl-(1, 2)-β-d-fructofuranose, known for its stabilizing properties, and methyl glucosyl-3,4-dehydro-apo-8-lycopenoate, which exhibits antioxidant activity. While the genus Planococcus is commonly associated with hydrocarbon degradation, it is also recognized for its secretion of biosurfactants and bioemulsifiers.	(19)
*Sporosarcina*	Biomineralization, using organism metabolism for mineral formation, is a promising technology. *Sporosarcina pasteurii*, known for efficient urea degradation, is a key focus for applications in construction and environmental protection. This review summarizes *S. pasteurii*’s features in biomineralization, discusses its progress in construction and environmental use, and addresses challenges in large-scale applications, providing insights for researchers.	(20)
*Turicibacter*	Turicibacter and Acidaminococcus are predictive indicators for immune-related adverse events and the effectiveness of immune checkpoint inhibitors.	(21)
*Streptococcus*	The more common Streptococcus is *Streptococcus suis*, which can cause porcine septicemia, meningitis, arthritis, and suppurative lymphadenitis; Streptococcus agalactis, Streptococcus aureus and Streptococcus mammary are chronic or recessive mastitis in cattle and sheep; *Streptococcus equi* is a subspecies that causes meningitis, bovine uterine inflammation, pneumonia and sheep septicemia; Streptococcus enterococcus can cause bovine catarrhal enteritis.	(22, 23)
*Bifidobacterium*	Bifidobacteria have a variety of probiotic functions, such as improving intestinal diseases caused by immune system disorders, such as inflammatory bowel disease, ulcerative colitis, Crohn’s disease, colonic pouch, and so on.	(24)
*Erwinia*	Erwinia is mainly parasitic on plants and causes spoilage. It is a kind of bacteria similar to *Escherichia coli* and an important part of non-plant pathogenic epiphytic microflora. Some Owens strains are conditional pathogens of human and other animals.	(25)

## Discussion

Microbial aerosols, constituting a complex colloidal system, encompass a diverse array of microbial components, including bacteria, viruses, Mycoplasma, Chlamydia, Rickettsia, exosomes, and more. These microorganisms are ubiquitous in nature and collectively form microbial aerosols. Scientific studies have established their pivotal role in the transmission of respiratory diseases, underscoring their significance in public health (11, 12, 26). Classified based on their primary components, microbial aerosols can be categorized into bacterial aerosols, fungal aerosols, and virus aerosols. Among these categories, bacterial and fungal aerosols are particularly noteworthy due to their substantial prevalence and impact on environmental and human health (27).

Exposure to microbial aerosols poses significant health risks due to their ability to infiltrate the body through various routes, including skin damage, mucosa, the respiratory tract, and the digestive tract. These microorganisms, once inside the body, can inflict irreparable damage on different bodily systems (28). Particularly concerning are airborne aerobic bacteria smaller than 2.0 μm, which can easily enter the body through the respiratory route. Upon inhalation, some of these bacteria deposit in the bronchi and bronchioles, disrupting the normal gas exchange in the lungs. Additionally, a portion of these microorganisms enters the circulatory system during the exchange of gases and blood, leading to even more severe health consequences for both humans and animals (29). Research by Bertrand has demonstrated that exposure to low concentrations of endotoxins, a component of microbial aerosols, can lead to increased lung inflammation (30). Moreover, exposure to microbial aerosols has been linked to the aggravation of chronic obstructive pulmonary disease (COPD), a debilitating respiratory condition (31). These findings underscore the detrimental impact of microbial aerosol exposure on respiratory health and highlight the importance of stringent measures to mitigate the risks associated with these airborne microorganisms. Therefore, understanding the intricacies of these microbial communities is crucial, as it not only provides insights into the ecological dynamics within animal environments but also informs strategies for disease prevention and control, safeguarding both animal populations and human communities.

In this study, the application of Illumina MiSeq sequencing technology marked a significant advancement in our comprehension of the bacterial community structure in sheep house air. This cutting-edge technology has ushered in a new era of research, allowing for a deeper and more intricate exploration of the microbial landscape within agricultural environments. Our sequencing results consisting of 11,207 OTUs, represents a rich diversity of microbial taxa, illuminating the intricate and multifaceted nature of the microbial ecosystem thriving within sheep pens. Alpha and beta diversity analyses are fundamental tools in microbial ecology, revealing vital insights into the complexity of microbial communities (32). Alpha diversity measures species diversity within individual samples, with the Chao1 index estimating species richness and the Shannon index quantifying abundance distribution. Higher values in YCSM samples indicate a diverse microbial community, likely due to various factors like animal health, genetics (33). Beta diversity assesses differences in species composition across samples, employing Principal Coordinate Analysis (PCoA) to visualize dissimilarities. PCoA reveals distinct clustering patterns among YCSH1, YCSH2, and YCSM farms. YCSH1 and YCSH2 share similarities, while YCSM displays a unique composition, possibly influenced by specific environmental factors on that farm.

At the taxonomic level, the prevalence of distinct microbial groups within the sheep house environment, including Firmicutes, Proteobacteria, Actinobacteria, Proteus, Actinomycetes, Bacillus, Psychrobacter, Acinetobacter, Streptococcus, and Bifidobacteria, discloses the existence of distinctive ecological niches (34). In the context of animal health, these phyla have been recognized for their diverse contributions to host physiology. Firmicutes, known for their ability to ferment complex carbohydrates, are implicated in energy extraction from dietary fibers, influencing the host’s metabolic processes (35). Proteobacteria, comprising numerous pathogenic and commensal species, can modulate immune responses and are integral to maintaining the microbial balance in the gut (36). Actinobacteria, characterized by their role in organic matter decomposition, contribute to nutrient cycling and may have immunomodulatory effects on the host (37). The intricate interplay between these bacterial phyla in the gastrointestinal tract underscores their significance in promoting digestion, nutrient absorption, and overall animal well-being. Bacillus, for instance, exerts inhibitory effects on various pathogens through the secretion of a multitude of antibacterial secondary metabolites, exemplified by *Bacillus licheniformis*. The antimicrobial repertoire of *Bacillus licheniformis* encompasses bacteriocins, which exhibit activity against Gram-positive or Gram-negative bacteria, fungal pathogens, and amoeba cells (38). Notably, pathogenic variants, such as zoonotic *Bacillus anthracis* and *Bacillus cereus*, are implicated in this microbial landscape (39). Psychrobacter, another prevalent genus, holds clinical significance, with certain strains causing human infections, including ocular infections, bacteremia, endocarditis, and meningitis (15). Acinetobacter is associated with a spectrum of clinical illnesses, including pneumonia, meningitis, peritonitis, endocarditis, and infections of the urinary tract and skin. Acinetobacter bacteremia poses a substantial and escalating challenge due to its high morbidity and mortality rate (40). Streptococcus demonstrates pathogenicity in various animal species, causing conditions such as porcine septicemia, meningitis, arthritis, suppurative lymphadenitis, chronic mastitis in cattle, sheep meningitis, bovine uterine inflammation, pneumonia, sheep septicemia, bovine catarrhal enteritis, fibrinous pleuritis, and fibrinous pericarditis (41–43). Bifidobacteria, on the other hand, manifest diverse probiotic functions, including the amelioration of intestinal diseases stemming from immune system disorders, such as inflammatory bowel disease and ulcerative colitis (24). Erwinia, predominantly parasitic on plants and contributing to spoilage, shares similarities with *Escherichia coli* and comprises strains that are conditional pathogens for humans and other animals (25). The Planococcaceae genus, a newly classified group, exhibits substantial phylogenetic overlap with other genera, particularly Bacillus, yet its specific functional role remains elusive (44).

Addressing the challenges posed by these pathogens necessitates multifaceted strategies, integrating scientific insights with practical interventions. The timely removal of fecal matter reduces potential contamination sources, while rigorous disinfection protocols curtail the proliferation of harmful microorganisms. Optimizing ventilation systems ensures the circulation of clean air, minimizing the risk of airborne pathogen transmission. Enhancing ventilation systems, incorporating routine air quality monitoring, and implementing sanitation protocols represent proactive measures farmers can take to mitigate the presence of harmful microbial aerosols (29). Furthermore, the identification of prevalent pathogenic bacteria offers valuable insights for implementing preventive strategies, including vaccination protocols or adjustments to animal husbandry practices, thereby minimizing the potential impact of these pathogens on sheep health. Future research can focus on the seasonal dynamics of microbial communities in sheep housing, explore correlations between environmental factors and microbial aerosols, and assess the effectiveness of interventions like ventilation and sanitation.

## Conclusion

This study showed that the bacterial concentration, species and diversity index of bacterial community were different in the three sheep houses. Acinetobacter, Streptococcus, Psychrophilic, Bacillus and Bifidobacterium were detected in three different sheep houses. These bacteria with potential risks to sheep and human health should be monitored. The disinfection frequency should be increased to 2–3 times a week or once every other day. One disinfectant should be replaced with another disinfectant after 2–3 weeks of continuous use, so as to avoid pathogenic microorganisms that produce drug resistance and reduce the disinfection effect. In this study, we highlighted the necessity of long-term detection of microbial aerosols in sheep houses and the importance of more comprehensive research and analysis of microbial aerosols in livestock houses.

## Data availability statement

The original contributions presented in the study are publicly available. This data can be found at: https://www.ncbi.nlm.nih.gov/bioproject/; PRJNA1048858.

## Ethics statement

The animal study was reviewed and approved by the Animal Care and Use Committee (NXKJLL-2022-041) of the Ningxia Society of Science and Technology Ethics (Ningxia, China). Written informed consent was obtained from the owners “Ningxia Tan sheep farm” to allow animals to participate in this study. The studies were conducted in accordance with the local legislation and institutional requirements. Written informed consent was obtained from the owners for the participation of their animals in this study.

## Author contributions

JW: Conceptualization, Formal analysis, Methodology, Project administration, Writing – original draft, Writing – review & editing. YY: Conceptualization, Formal analysis, Writing – original draft, Writing – review & editing. AR: Methodology, Writing – original draft, Writing – review & editing. YG: Conceptualization, Writing – original draft, Writing – review & editing. QM: Methodology, Writing – original draft, Writing – review & editing. DL: Formal analysis, Writing – original draft, Writing – review & editing.
