# Supplementary figures and images for: The distribution characteristics of aerosol bacteria in different types of sheepfolds
Source: Front Vet Sci. 2024 Feb 13;11:1348850. doi: 10.3389/fvets.2024.1348850 (PMC10900508; doi:10.3389/fvets.2024.1348850)

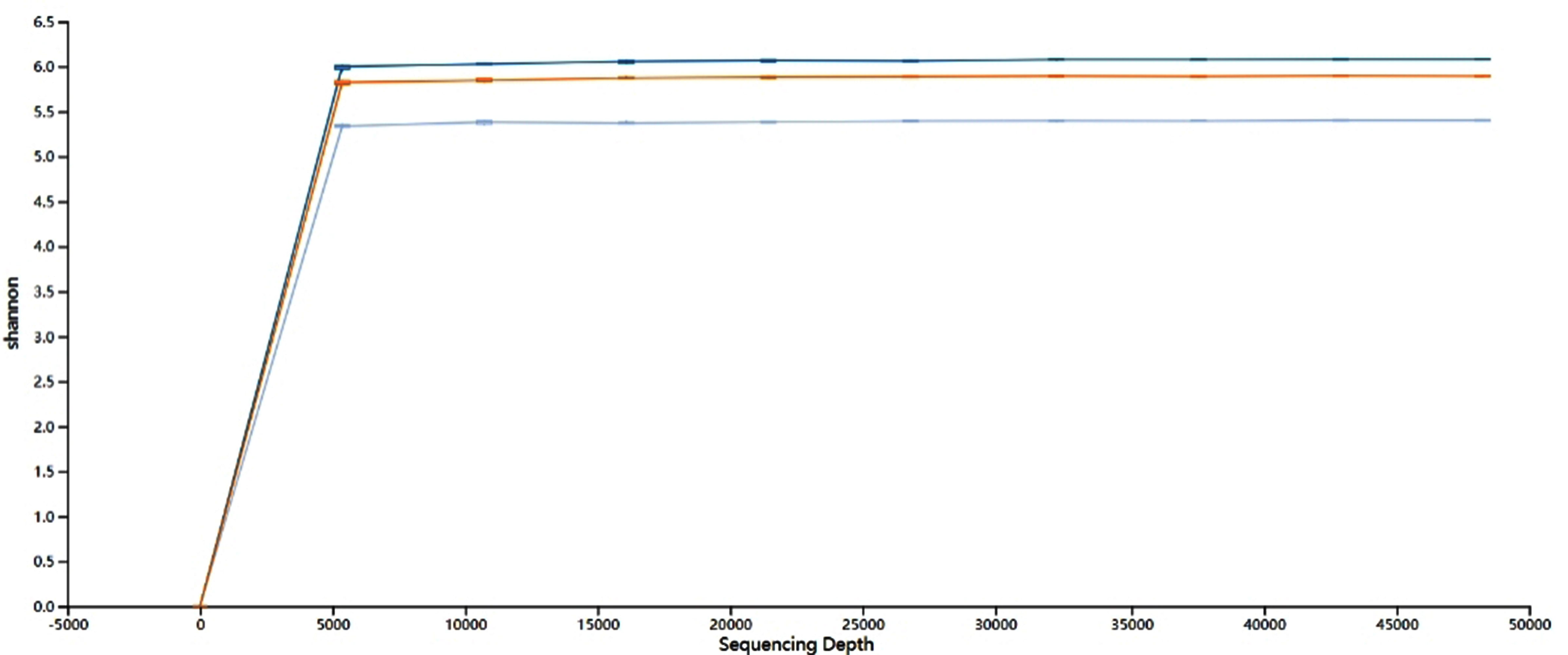

Supplement: Supplementary file 1 [file Image_1.JPEG]
